# Direct Coculture of Human Chondrocytes and Synovium-Derived Stem Cells Enhances *In Vitro* Chondrogenesis 

**DOI:** 10.22074/cellj.2018.5025

**Published:** 2018-01-01

**Authors:** Tae Woo Kim, Myung Chul Lee, Hyun Cheol Bae, Hyuk-Soo Han

**Affiliations:** 1 Department of Orthopaedic Surgery, Hallym University Chuncheon Sacred Heart Hospital 77, Sakju-ro, Chuncheon-si, Gangwon-do, Korea; 2 Department of Orthopaedic Surgery, Seoul National University Hospital 101 Daehang-ro, Jongno-gu, Seoul, Korea

**Keywords:** Chondrocyte, Chondrogenesis, Coculture, Mesenchymal Stem Cells, Synovium

## Abstract

**Objective:**

Coculture of chondrocytes and mesenchymal stem cells (MSCs) has been developed as a strategy to overcome the dedifferentiation of chondrocytes during *in vitro* expansion in autologous chondrocyte transplantation. Synovium-derived stem cells (SDSCs) can be a promising cell source for coculture due to their superior chondrogenic potential compared to other MSCs and easy accessibility without donor site morbidity. However, studies on coculture of chondrocytes and SDSCs are very limited. The aim of this study was to investigate whether direct coculture of human chondrocytes and SDSCs could enhance chondrogenesis compared to monoculture of each cell.

**Materials and Methods:**

In this experimental study, passage 2 chondrocytes and SDSCs were directly cocultured using different ratios of chondrocytes to SDSCs (3:1, 1:1, or 1:3). glycosaminoglycan (GAG) synthetic activity was assessed using GAG assays and Safranin-O staining. Expression of chondrogenesis-related genes (*collagen types I, II, X, Aggrecan,* and *Sox-9*) were analyzed by reverse transcription-quantitative polymerase chain reaction (RT-qPCR) and immunohistochemistry staining.

**Results:**

GAG/DNA ratios in 1:1 and 1:3 coculture groups were significantly increased compared to those in the chondrocyte and SDSC monoculture groups. *Type II collagen* and *SOX-9* were significantly upregulated in the 1:1 coculture group compared to those in the chondrocyte and SDSC monoculture groups. On the other hand, osteogenic marker (type I collagen) and hypertrophic marker (type X collagen) were significantly downregulated in the coculture groups compared to those in the SDSC monoculture group.

**Conclusion:**

Direct coculture of human chondrocytes and SDSCs significantly enhanced chondrogenic potential, especially at a ratio of 1:1, compared to chondrocyte or SDSC monocultures.

## Introduction

Cartilage has limited capacity for intrinsic healing due to its avascularity and low chondrocyte regeneration rate. Despite various efforts to treat cartilage injury, innate repair of cartilage tissue remains a challenging issue. Cell based therapies have been developed to overcome the poor healing potential of cartilage. Autologous chondrocyte transplantation (ACT) which consists of chondrocyte harvest, *in vitro* chondrocyte expansion, and implantation of cultivated chondrocytes, has been introduced as a promising cell based treatment for cartilage repair (1, 2). However, dedifferentiation of chondrocytes during *in vitro* expansion decreases the chondrogenic phenotype, resulting in the production of repair tissue whose mechanical properties are inferior to those of hyaline cartilage (3, 4). 

To overcome the limitations of current ACT techniques and improve clinical outcomes, various efforts to enhance chondrogenesis of articular cartilage have been performed. The strategy of using coculture has been developed to enhance the chondrogenic phenotype of chondrocytes and mesenchymal stem cells (MSCs) as used in tissue engineering for cartilage repair. Coculture of these two cell types synergistically promotes the redifferentiation of chondrocytes and increases the chondrogenic differentiation of MSCs during *in vitro* expansion, resulting in enhanced chondrogenesis (5-11). Numerous studies have been performed over the last decade on cocultures involving various kinds of MSCs such as bone marrow, umbilical cord blood, adipose tissues, and synovium. Among these tissues, synovium-derived stem cells (SDSCs) are known to possess chondrogenic potential superior to that of MSCs derived from other tissues. However, very few coculture studies of chondrocytes and SDSCs have been reported. 

Wang et al. have shown that the coculture of SDSCs and *TGF-b3* gene transfected chondrocytes can improve chondrogenesis in direct coculture as well as in indirect coculture (12, 13). However, these studies were performed using SDSCs and chondrocytes from animals, such as rabbits or pigs. Kubosch et al. (14) have reported that indirect coculture of human SDSCs and chondrocytes can enhance the chondrogenic phenotype of SDSCs through a paracrine effect on the cocultured chondrocytes. However, cell to cell interaction between human SDSCs and chondrocytes cannot be evaluated in this type of indirect coculture setting. 

The purpose of this study was to investigate whether direct mixed coculture of human chondrocytes and SDSCs could enhance chodrogenesis compared to monoculture of the SDSCs or chondrocytes. As far as we know, this is the first study to investigate this. Three different ratios of the two cell types were evaluated to determine the ideal ratio for direct coculture. It is anticipated that results from studies of the direct coculture of SDSCs and chondrocytes might be used in the next generation ACT and MSC-based therapies for the treatment of cartilage injury. 

## Materials and Methods

### Harvest of synovium and cartilage tissue 

In this experimental study, synovium and cartilage tissues were obtained from six female osteoarthritis patients (age 66 to 72 years) undergoing total knee arthroplasty (TKA). In all patients, the Kellgren Lawrence grade was 4 and osteoarthritis had progressed at the medial side of knee. For this reason the study was performed using relatively intact cartilage from the lateral femoral and tibial condyles. Synovium was harvested from the suprapatellar pouch. Ethical approval for this study was obtained from Seoul National University Boramae Medical Center Institutional Review Board (06-2012-25). Those who had inflammatory arthritis, prior knee joint infection, and intraarticular trauma were excluded.

### Isolation of synovium-derived stem cells

Synovial tissue was minced in phosphate-buffered saline (PBS) and digested with 0.02% collagenase (Sigma, St. Louis, Missouri) overnight. Cells were filtered from undigested tissue with 70 µm sieves and centrifuged at 1,500 rpm for 5 minutes. Then, cells were cultured in low glucose Dulbecco’s modified Eagle’s medium (LG-DMEM, Gibco, UK) with 10% fetal bovine serum (FBS) and 1% penicillin/ streptomycin/amphotericin at 37°C with 5% CO_2_. The medium was changed after 48 hours and nonadherent cells were removed during this procedure. Passage 2 cells were used in the pellet coculture. 

### Isolation of chondrocytes 

Cartilage was digested at 3°C with 0.2% pronase (Sigma, Germany) for 1 hour and with 0.2% collagenase (Sigma, Germany) overnight. Cells were filtered from undigested tissue with 70 µm sieves and centrifuged at 1,500 rpm for 5 minutes. Subsequently collected chondrocytes were cultured in LG-DMEM with 10% FBS and 1% penicillin/streptomycin/amphotericin at 37°C with 5% CO_2_ and expanded on culture dishes at a density of 1×10^6^ /150 ml. The initial number of chondrocytes from each patient ranged from 1.5×10^6^ to 3×10^6^. Passage 2 cells were used in the pellet cocultures.

### Mixed coculture of chondrocytes and synoviumderived stem cells 

In our previous study, we observed no change in the chondrogenic phenotype of SDSCs after the passage 1 period (15). In addition, the initial number of SDSCs and chondrocytes obtained from the harvested synovium and cartilage was not sufficient for the experiment. Therefore, passage 2 chondrocytes and SDSCs were used for the pellet cocultures. Five groups of passage 2 cell suspensions containing 5×10^5^ chondrocytes or SDSCs, or a combination of chondrocytes and SDSCs in three different ratios (Table 1) were centrifuged at 1,500 rpm for 5 minutes to obtain cell pellets. Cell pellets were cultured in chondrogenic medium (LG-DMEM) containing 0.1 mmol/L ascorbic acid 2-phosphate, 100 nmol dexamethasone, 40 g/mL proline, 100 U/mL penicillin, 100 g/mL streptomycin, and ITS Premix (BD Biosciences, Massachusetts) supplemented with transforming growth factor beta 1 (TGF-ß1). The culture medium was changes every other day until day 21. Chondrogenesis of the cell pellets was evaluated at days 7, 14, and 21 (16).

**Table 1:** Coculture ratio and cell counts for the five cell pellet culture groups


	Chondrocyte	Coculture 1	Coculture 2	Coculture 3	SDSC

Ratio (chondrocyte: SDSC)	4:0	3:1	1:1	1:3	0:4
Cell count (cells) (chondrocyte: SDSC)	5×10^5^	3.75×10^5^	1.25×105	1.25×10^5^	5×10^5^
		1.25×10^5^	2.5×10^5^	3.75×10^5^	


SDSC; Synovium-derived stem cell.

### Histology and immunohistochemistry 

For histological evaluation of glycosaminoglycan (GAG) synthesis, cell pellets from each group were stained with Safranin-O and fast green staining at days 7, 14, and 21. Staining was performed as described in our previous study (17). The staining was graded using the Bern Score, developed to evaluate Safranin-O staining via three different measures; uniformity and darkness, distance between cells, and cell morphologies (18). To evaluate the production of type II and X collagen histologically, immunohistochemical staining was performed in each group at days 7, 14, and 21 using mouse anti-human monoclonal antibodies for type II and X collagen (Neomarkers, California). Staining of type II and X collagen was examined separately and detail procedures were performed as described previously in our study (17). In the interpretation of the immunohistochemical results, synthesis of type II and X collagen was evaluated by brown staining compared to background blue-purple color.

### Biochemical analysis 

To assess glycosaminoglycan synthesis, total GAG and DNA were measured. GAG levels were evaluated with dimethylmethylene blue (DMB) (19). Cell pellets from each group were collected in two different fractions (matrix and media) at day 21. Cell pellets were digested in papain buffer (5 mM L-cysteine, 200 µg/ml papain, 0.1 M sodium acetate, pH=3.0) for 18 hours at 65°C and centrifuged for 5 minutes at 6,000 rpm. Subsequently, aggregated cells were placed in 96 well plates with DMB solution. GAG levels were determined by absorbances measured at 530 and 590 nm using an immunoassay reader. The absorbance value was standardized using chondroitin-6-sulfate. The DNA content of pellets was measured using a Quant-iT PicoGreen dsDNA Assay Kit (Invitrogen, Oregan) (5). GAG synthetic activity was assessed from total GAG content normalized by total DNA content. 

### Reverse transcription-quantitative polymerase chain reaction 

At culture day 21, expression of chondrogenesisrelated genes including *Aggrecan*, Sry-type highmobility- group box transcription factor-9 *(Sox9), Type I collagen, Type II collagen* and *Type X collagen* was evaluated using reverse transcription- quantitative polymer chain reaction (RT-qPCR). Total RNA was purified from cell pellets using TRIzol reagent (Invitrogen) and complementary DNA was prepared with RNA to cDNA EcoDry™ Premix (Oligo dT) and cDNA Synthesis Kit (Takara Bio, Japan). Primer Express software version 1.5 (Abingdon, UK) was used for the analytic procedure during RT-qPCR and the level of glyceraldehyde3- phosphate dehydrogenase (*GAPDH*) was used as an endogenous reference. Relative quantification of gene expression was performed using the ABI Prism 7000 Sequence Detection System with the relative standard curve method (20). 

### Statistical analysis 

Statistical analysis was performed using SPSS 18.0 software (SPSS, Chicago, IL). Kruskal-Wallis tests were used to compare GAG synthetic activity, Bern score, and gene expression among the 5 culture groups. Intergroup differences were assessed using the Mann-Whitney test. Findings were considered statistically significant when the Pvalue was less than 0.05.

## Results

### Cellularity and glycosaminoglycan synthesis

**Fig.1:**
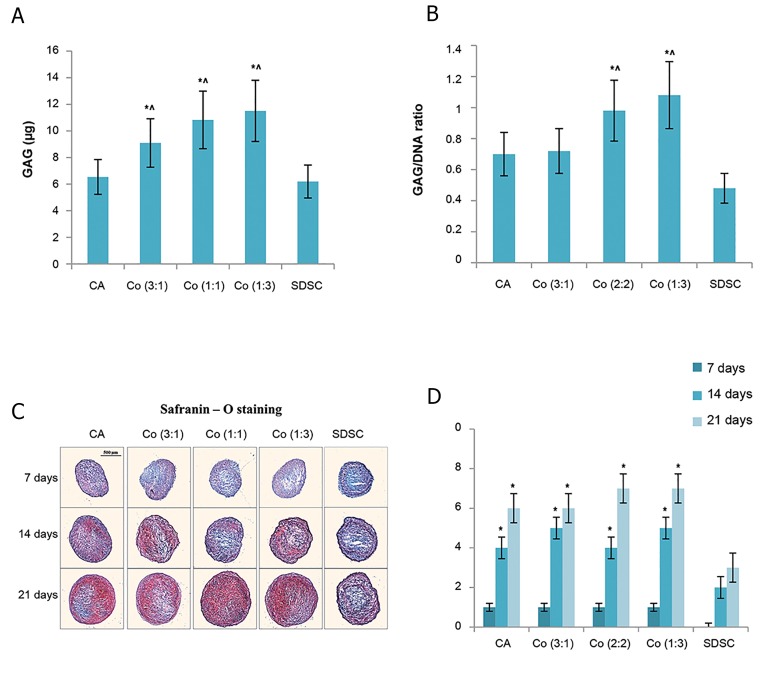
GAG production in pellet culture. A, B. Evaluation of GAG synthetic activity (total GAG content and GAG/DNA ratio), C. Histological evaluation of GAG production with Safranin-O staining, and D. Histological scoring (Bern Score). Results are presented as mean ± SD (n=6). *, ^; Significant difference compared to SDSCs and chondrocyte groups, respectively (P<0.05), GAG; Glycosaminoglycan, CA; Chondrocyte, Co; Coculture,and SDSC; Synovium-derived mesenchymal stem cell.

Total cellular DNA and GAG depositions were measured at day 21. There was no significant difference in total DNA content among the five culture groups. However, GAG content was significantly increased in the 3:1, 1:1, and 1:3 coculture groups compared to that in either the chondrocyte or SDSC monoculture group. The 1:3 coculture group showed the highest GAG activity among the three coculture groups. The 1:1 and 1:3 coculture groups had significantly higher GAG/ DNA ratios than the chondrocyte or the SDSC monoculture group (Fig .1A). 

### Histological analysis 

Presence of proteoglycans was evaluated with Safranin O-fast green staining on days 7, 14, and 21 for all five groups (Fig .1B). On day 7, weak staining was observed in the chondrocyte monoculture and the three coculture groups. However, staining was not observed in the SDSC monoculture group. On day 21, dense and even staining was observed in the 1:1 and 1:3 coculture groups. Partial staining was observed in the 3:1coculture group and the chondrocyte monoculture group. Staining in the SDSC monoculture group was very weak. Safranin O-fast green staining was also evaluated using the Bern Score which is known to be significantly correlated with GAG content (18). On day 21, Bern scores for the chondrocyte monoculture group and the three coculture groups were significantly higher than those for the SDSC group (Fig .1C). Overall, the histological findings matched well with the results of GAG/DNA assay. 

### Gene expression analysis using polymerase chain reaction

**Fig.2:**
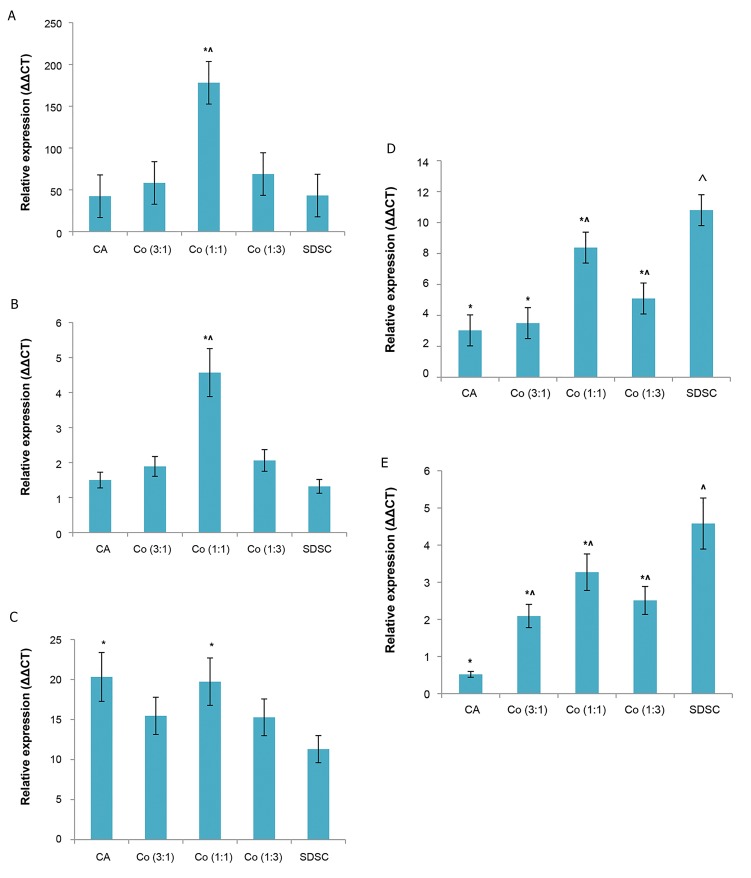
RT-PCR analysis for chondrogenesis-related gene expression after 21 days of culture. Results are presented as mean ± SD (n=6). ***A.** Col II, **B.** Sox-9, **C.** Aggrecan, **D.** Col I, **E.** Col X.* *, ^; Significant difference compared to SDSC group and chondrocyte group, respectively (P<0.05), RT-PCR; Reverse transcription-polymerase chain reaction, CA; Chondrocyte, Co; Coculture, and SDSC; Synovium- derived stem cell.

Chondrogenesis-related gene expression was quantified with qRT-PCR at day 21 (Fig .2). *Type II collagen, Aggrecan,* and *Sox-9* were evaluated as chondrogenic markers. Levels of *Type II collagen* and *Sox-9* in the 1:1 coculture group were significantly higher than those in the 1:3 and 3:1 coculture groups as well as in the chondrocyte and SDSC monoculture groups. Expression levels of *Aggrecan* in the chondrocyte monoculture and 1:1 coculture groups were significantly increased compared to those in the SDSC monoculture group. However, there was no statistical difference in the expression of *Aggrecan* between the 1:3 and 3:1 coculture groups, or the SDSC monoculture group.

To assess dedifferentiation of chondrocytes and osteogenic induction of SDSC, levels of Type I collagen were evaluated. Type I collagen levels in the chondrocyte monoculture and the three coculture groups were significantly lower than those in the SDSC monoculture group during the 21-day culture period. However, the 1:1 coculture group showed a significantly higher level of Type I collagen compared to the chondrocyte monoculture group. To exclude hypertrophic change during chondrogenesis, Type X collagen was evaluated as a hypertrophic marker. As expected, the levels of Type X collagen in the three coculture groups were significantly lower than in the SDSC monoculture group and higher than in the chondrocyte monoculture group.

### Immunohistochemical analysis 

**Fig.3:**
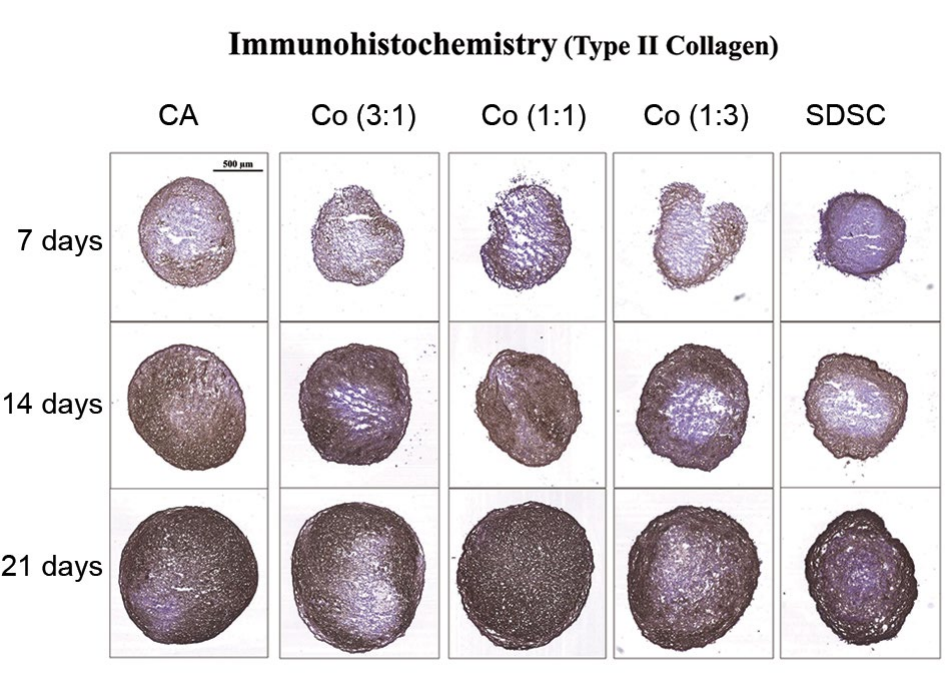
Immunohistochemistry for type II collagen chondrogenic marker. Staining on day 21 was the most prominent in the 1:1 ratio coculture group. CA; Chondrocyte, SDSC; Synovium derived stem cell, and Co; Coculture.

**Fig.4:**
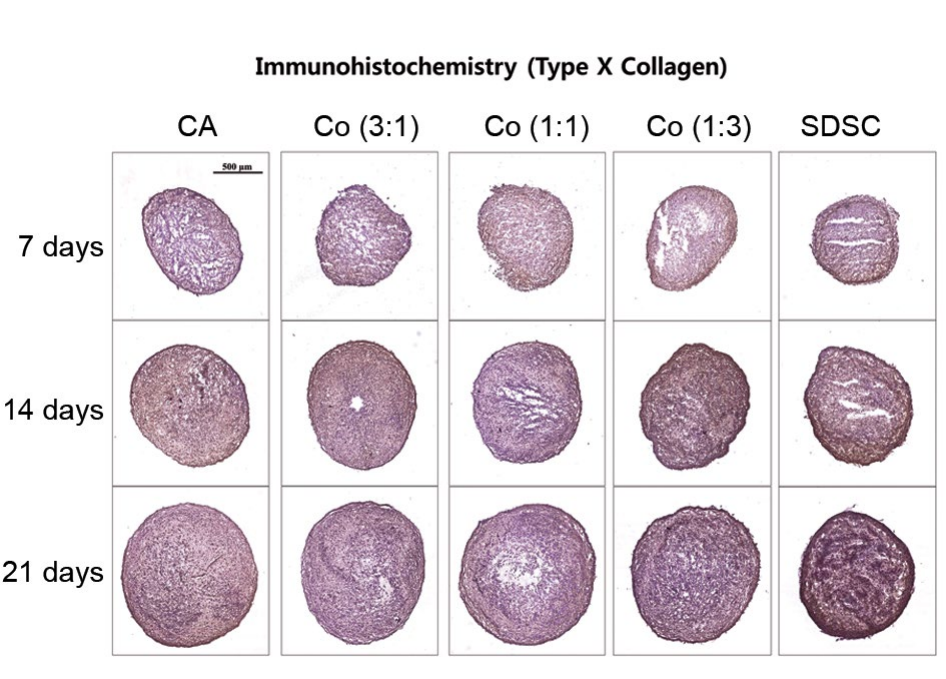
Immunohistochemistry for type X collagen hypertrophic marker. Staining in the SDSC group was prominent compared to that in the three coculture groups on day 21. CA; Chondrocyte, SDSC; Synovium-derived stem cell, and Co; Coculture.

Immunohistochemistry was performed for type II and type X collagen as the representative chondrogenic and hypertrophic markers in chondrogenesis, respectively. Staining of type II collagen was similar among the three coculture groups on day 7 (Fig .3). However, the most dense and homogeneous staining was observed D in the 1:1 coculture group on day 21. On the other hand, staining of type X collagen was most prominent in the SDSC monoculture group (Fig .4) on day 21, with only slight staining observed in the chondrocyte monoculture group and the three coculture groups. Immunohistochemistry staining for type II and X collagens correlated well with the gene expression results based on qRT-PCR. 

## Discussion

Coculture of chondrocytes and MSCs has been presented as a solution to improving autologous chondrocyte transplantation because the chondrogenic phenotype of the chondrocytes can be maintained during *in vitro* expansion. In addition, the amount of cartilage required for *in vitro* culture can be reduced proportional to the amount of MSCs, resulting in a decrease in donor site morbidity. SDSCs have been reported to possess superior chondrogenic potential to other MSCs and are known to be tissue-specific for cartilage engineering. Also, synovium can be obtained arthroscopically with minimum invasiveness during the cartilage harvest procedure (21). Therefore, additional procedures for tissue harvest are unnecessary and complications, such as the pain and hematoma associated with harvesting bone marrow-derived MSCs (BM-MSCs), can be avoided (22). However, whether the direct coculture of human chondrocyte and SDSCs can enhance chondrogenesis with reduced hypertrophy has not been proved unequivocally.

In the present study, direct coculture of human chondrocytes and SDSCs enhanced chondrogenesis compared to the monoculture of chondrocyte or SDSCs. Three coculture ratios of chondrocytes and SDSCs were evaluated (3:1, 1:1, and 1:3) to find the optimal ratio for chondrogenesis. Results from the GAG assay revealed that GAG synthetic activities in the 1:1 and 1:3 coculture groups were significantly higher compared to those in the chondrocyte and SDSC monoculture groups. The 1:3 coculture group had the highest GAG synthetic activity among the three coculture groups. These findings were very similar to the results of a coculutre study by Lai et al. (23) using human chondrocytes from patients undergoing total knee arthroplasty (TKA) and adipose derived stem cells. Their results showed the coculture groups to have superior GAG synthetic activities to the SDSC or chondrocyte monoculture groups, especially at ratios of 1:1 and 1:3. On the other hand, GAG activity of the chondrocyte group was comparable to that of coculture groups in the study of Meretoja et al. (5) using bovine primary chondrocytes and BM-MSCs. We assume that the chondrogenic potential of chondrocyte or coculture groups can be affected by donor age or cell passage of chondrocytes and MSCs (24, 25).

Gene expression analysis revealed that the levels of *Type II collagen* and *Sox-9* were significantly increased in the 1:1 coculture group compared to those in chondrocyte and SDSC monoculture groups. However, expression levels for *Aggrecan* were similar between the chondrocyte monoculture group and the 1:1 coculture group. The low level of type II collagen in the chondrocyte group can be interpreted in the same way as the low GAG activity in the chondrocyte group above. Overall, the levels of chondrogenesis-related genes were upregulated in the 1:1 coculture group compared to those in other groups. On the other hand, the level of type I collagen in the SDSC monoculture group was significantly increased compared to that of 1:3 and 3:1 coculture groups. The relatively higher level of type I collagen in the 1:1 coculture group is probably associated with highly expressed type II collagen. However, the exact cellular mechanism behind this finding is not clear and further studies, including changes in fibroblasts after coculture seem to be necessary. The expression of collagen type I and type II and aggrecan in this study were similar to those in the coculture study of Lai et al. (23), except that the 1:3 coculture group also showed comparable chondrogenic potential to the 1:1 coculture group in their study. 

The difference between adipose derived MSCs and synovium derived MSCs in the two studies might have affected the optimal coculture ratio. In the majority of previous direct coculture studies that have used various coculture ratios the optimal ratio of chondrocytes to BM-MSCs or adipose-derived MSCs ranged from 25 to 50% (5, 23, 26). However, to our knowledge, there has been no previous coculture study of different ratios of chondrocytes and SDSCs. In this study, the optimal ratio for the coculture of chondrocytes and SDSCs was found to be from 25 to 50% of chondrocytes, similar to coculture studies using BM-MSCs or adipose-derived MSCs.

Another remarkable finding of this study was the decrease in type X collagen, a hypertrophic marker, in the coculture groups compared to that in the SDSC monoculture group. MSCs can express a hypertrophic phenotype under chondrogenic induction, resulting in calcification of the extracellular matrix, which (27) can limit their clinical application to the treatment of cartilage injury. Some authors suggest that type X collagen is not an ideal hypertrophic marker for MSCs because it can increase before MSCs differentiate into chongrogenic cells (28). However, early expression of type X collagen was not observed in our coculture study, and various other coculture studies have evaluated MSC hypertrophy using type X collagen. Cooke et al. (9) and Glovannini et al. (10) have reported that coculture of chondrocyte and bone marrow derived MSCs can reduce the expression of type X collagen. Decreased hypertrophy of adipose derived MSC has also been observed in the coculture study of Lee and Im (29). However, although the potential of the coculture of SDSCs and chondrocytes to reduce hypertrophy in the SDSCs has not yet been investigated, results of the present study can be used as a basis for the clinical use of SDSC in hypertrophy prevention.

The exact cellular mechanism underlying the enhanced chondrogenesis observed in direct coculture remains unclear. Some studies have suggested that MSC differentiation is essential to the chondrogenic mechanism following direct coculture (8, 29). On the other hand, Wu et al. (26) have reported that MSCs can stimulate cartilage formation due to a trophic effect on chondrocytes rather than differentiating into chondrocytes in coculture pellets. In the present study, a chondrogenic phenotype was expressed in both the chondrocyte and SDSC monocultures. This leads us to suggest that chondrogenesis in direct coculture is achieved by the synergism of chondrocyte redifferentiation and chondrogenic differentiation of the SDSCs. Although the exact contribution of each cell cannot be determined, it is clear that a combination of human chondrocytes and SDSCs can enhance chondrogenesis, and that this combination can be a good cell source to overcome the limitations of current ACT treatment such as dedifferentiation of chondrocytes during *in vitro* expansion. 

A limitation of the current study is that the human chondrocytes and SDSCs investigated in this study were obtained from old female patients undergoing total knee arthroplasty. It has been reported that the proliferation and chondrogenic potential of chondrocytes can be influenced by donor age (24). Considering that autologous chondrocyte transplantation is recommended for patients under 45-50 years old, chondrocytes from TKA might be a less than ideal source of cells. However, it is not easy to obtain healthy cartilage from young donors for ethical reasons, which may be why several coculture studies have also obtained human chondrocytes from arthroplasty surgery (14, 30, 31). On the other hand, Kubosch et al’s (32) recent study showed that the expression level of type II collagen in SDSCs was not affected by age and arthritis of donor. Although donor age might be a limitation, this study demonstrated meaningful comparison of chondrogenic potential among chondrocyte and SDSC coculture groups and monocultures of each cell type.

## Conclusion

Overall, the coculture of human chondrocytes and SDSCs showed enhanced chondrogenic potential compared to the monoculture of either cell type, especially in coculture at a ratio of 1:1. In addition, the levels of type I and type X collagen in the coculture groups were significantly reduced compared to those in the SDSC monoculture group. We conclude that the direct coculture of human chondrocyte and SDSCs could be a useful strategy to improve the outcome of current autologous chondrocyte transplantation treatment. 
